# The experiences of postnatal women and healthcare professionals of a brief weight management intervention embedded within the national child immunisation programme

**DOI:** 10.1186/s12884-021-03905-3

**Published:** 2021-06-29

**Authors:** Natalie Tyldesley-Marshall, Sheila M Greenfield, Helen M Parretti, Kate Jolly, Susan Jebb, Amanda J Daley

**Affiliations:** 1 School of Sport, Exercise and Health Sciences, Loughborough University, Loughborough, Leicestershire LE11 3TU UK; 2 Institute for Applied Health Research, University of Birmingham, Birmingham, Edgbaston B15 2TT UK; 3 Norwich Medical School, University of East Anglia, Norwich, Norfolk NR4 7TJ UK; 4 Nuffield Department of Primary Care Health Sciences, University of Oxford, Oxford, OX2 6GG UK; 5 The Centre for Lifestyle Medicine and Behaviour, School of Sport, Exercise and Health Sciences, Loughborough University, Loughborough, Leicestershire LE11 3TU UK

**Keywords:** Obesity, Postnatal, Weight loss, Qualitative, Practice nurses, Child immunisations

## Abstract

**Background:**

After childbirth, most women do not lose the extra weight gained during pregnancy. This is important because postnatal weight retention contributes to the development of obesity in later life. Research shows that postnatal women living with overweight would prefer to weigh less, are interested in implementing weight loss strategies, and would like support. Without evidence for the benefit of weight management interventions during pregnancy, postnatal interventions are increasingly important. Research has focused on intensive weight loss programmes, which cannot be offered to all postnatal women. Instead, we investigated the feasibility of a brief intervention delivered to postnatal women at child immunisation appointments. This qualitative study explored the views of women who received the intervention and healthcare professionals who delivered it.

**Methods:**

The intervention was delivered within the context of the national child immunisation programme. The intervention group were offered brief support encouraging self-management of weight when attending general practices to have their child immunised at two, three and four months of age. The intervention involved motivation and support from practice nurses to encourage women to make healthier lifestyle choices through self-monitoring of weight and signposting to an online weight management programme. Nurses provided external accountability for weight loss. Women were asked to weigh themselves weekly and record this on a weight record card. Nested within this trial, semi-structured interviews explored the experiences of postnatal women who received the intervention and nurses who delivered it.

**Results:**

The intervention was generally acceptable to participants and child immunisation appointments considered a suitable intervention setting. Nurses were hesitant to discuss maternal weight, viewing the postnatal period as a vulnerable time. Whilst some caveats to implementation were discussed by nurses, they felt the intervention was easy to deliver and would motivate postnatal women to lose weight.

**Conclusions:**

Participants were keen to lose weight after childbirth. Overall, they reported that the intervention was acceptable, convenient, and, appreciated support to lose weight after childbirth. Although nurses, expressed concerns about raising the topic of weight in the early postnatal period, they felt the intervention was easy to deliver and would help to motivate women to lose weight.

**Supplementary Information:**

The online version contains supplementary material available at 10.1186/s12884-021-03905-3.

## Background

The rising prevalence of obesity in women, combined with excess weight gain during pregnancy means that there are more women with obesity in the postnatal period [1–3]. This can have adverse health impacts for women in later life and increases health risks during subsequent pregnancies [4]. Postnatal women living with overweight would prefer to weigh less and would like support to achieve this, but little support is currently offered by the National Health Service (NHS) [5–7]. Evidence indicates that lifestyle interventions can significantly lower body weight in women who have given birth relative to comparators, although many of the interventions tested to date have been intensive and tailored lifestyle-based programmes usually delivered by healthcare professionals (HCPs) trained in weight management (e.g. psychologists and dieticians) [8]. Despite evidence suggesting that some of these interventions can be effective for postnatal women, the NHS lacks the workforce to scale-up these intensive interventions to make them available to all 820,000 women who give birth annually in the United Kingdom (UK), 520,500 of whom will be overweight at the start of each pregnancy [9].

One way of offering weight management interventions at scale is for HCPs to offer brief interventions embedded within existing universally delivered healthcare consultations. This type of approach to intervention could help to address many of the concerns that HCPs have about raising the topic of weight, by ‘normalising’ the topic, and it also addresses health inequalities [10, 11]. A randomised controlled cluster trial (RCT) to test the feasibility and acceptability of a brief weight loss intervention for postnatal women delivered by practice nurses and general practitioners (GPs) embedded within child immunisation appointment, relative to usual care was conducted [12, 13]. The within group results showed that, on average, intervention group lost weight − 3.3 kg, while the usual care group gained 1.9 kg over the three-four month intervention period. The trial protocol and findings have been published [12, 13]. This report aims to understand the experiences of postnatal women who received this weight loss intervention and the practice nurses who delivered it to them. The specific objectives for this study were to: explore whether child immunisation appointments were an appropriate setting to monitor and offer feedback on the weight to postnatal women; capture participants’ views about the usefulness of the intervention in helping them to manage their weight, including which intervention components they valued the most; to investigate HCPs feelings about raising the topic of weight with postnatal women at child immunisation appointments; capture HCPs views about delivering the intervention and the impact it had on child immunisation appointments; and gain feedback from HCPs about how to improve intervention delivery and content.

## Methods

### Design of the study

The study was granted favourable ethical opinion by the Black Country Research Ethics Committee (17/WM/0399). The results from the interviews with trial participants, practice nurses and a GP (herein referred to as nurses) have been presented together here to allow for cross comparisons of themes and learning from different experiences and perspectives. We aimed to gain a thorough understanding of, and be able to, describe trial participants and nurses’ experiences of an intervention embedded within an existing service. We therefore used a ‘generic approach’ to reflect this and did not set out to design the research using any specific theoretical perspective [14]. The Consolidated Criteria for Reporting Qualitative Research (COREQ) was used for reporting this study [15].

Purposive sampling was used as a nurse/GP and participant from each practice were recruited to explore consistency and variation in intervention delivery [16]. The interview schedules were developed specifically for this research study by the investigators (see Additional File 1). All interviews were conducted by the first author (female Research Fellow) with many years’ experience in sociological research (and a Masters in Social Research).

### Intervention

The trial intervention was deliberately developed to meet the ambition of the NHS to ‘Make Every Contact Count’ [17]. In the trial intervention group, when participants attended their practice for their first three child immunisation appointments, the nurse measured their weight and recorded this on an additional inserted page in the child health red book (a handheld record of child health issued at birth). Nurses also asked participants to self-monitor their weight weekly and record this on a record card attached inside the child record book. Women were also signposted to use an online weight loss programme (POWeR) [18] for weight loss support and advised to aim to lose 0.5 to 1 kg per week in line with current guidance [19]. The role of HCPs in this study was to provide a sense of external accountability to participants, the belief that someone other than oneself is observing and cares whether certain behaviours (self-weighing and losing weight) are adhered to. A full description of the trial has been published previously [12, 13].

### Participants in the trial intervention

Women aged at least 18 years old with a body mass index (BMI) ≥ 25 kg/m^2^ at trial baseline, and who had given birth at least four weeks prior to consent were eligible to participate in this study. Intervention group participants who had indicated a willingness to complete a semi-structured interview were contacted by telephone or email by the first author and provided with a verbal overview of the study purpose and procedures. Written informed consent was obtained. The semi-structured interviews were conducted one-on-one in participants’ homes after they had completed the trial (approximately 4-months postnatal), between January-July 2019. The interview schedule was aligned with the study objectives (Additional File 1). Each participant was interviewed once and had not met the interviewer prior to the interview. Interviews lasted between 14 and 45 min (median length 34 min). A £20 shopping voucher was offered to participants.

### Health care professionals who delivered the intervention

 Nurses were contacted by telephone or email once they had finished delivering the intervention to all the participants at their practice, to ensure the interview did not impact on their delivery of the intervention. Nurses were interviewed once and had not met the interviewer prior to the interview (Additional File 1 for the interview schedule). Written informed consent was taken prior to the face-to-face interviews, which were undertaken one-to-one. Verbal informed consent by telephone was obtained for the telephone interviews; at the beginning of the audio recordings nurses stated that they consented to take part. The interviews were conducted between May and June 2019 and lasted between 24 and 67 min (median length 34 min) (note many of these interviews were conducted over the telephone resulting in naturally shorter interview durations, however one nurse declined for the interview to be recorded, therefore handwritten notes were taken extending the interview length). Nurses received a £10 high shopping voucher for participating in the study.

### Data analysis

The interviews were digitally recorded using an encrypted audio recording device, and transcribed verbatim by a commercial transcription company (except the one interview with a nurse where handwritten notes were taken). Fieldnotes were taken immediately after the interviews and integrated into the transcripts. Transcripts were analysed using the Framework Method to allow a more systematic approach to reviewing, comparing, and searching for patterns in the data; as well as to provide more transparency in the coding and analysis process [20]. Whilst the trial participant interviews were analysed and completed first, the analyses were conducted concurrently and ideas and concepts from both sets of interviews informed the other. Interviews were checked and listened to repeatedly to allow the researcher to become familiar with the raw data. NTM examined transcripts line-by-line and assigned codes (derived from the data), then assigned to themes. An early transcript was independently reviewed by four authors (with different disciplinary backgrounds: sociology, psychology, general practice) (NTM, SG, AD, HP) to develop the working analytical framework. The team discussed subsequent codes and ideas to achieve consensus that would improve both the quality and rigour of the study. A pragmatic approach to data analysis was taken where the focus was on the key themes that would specifically contribute to the refinement of the intervention and the design of any future definitive trial.

Early thoughts about coding, themes and the direction of the analysis were also made and kept for increased transparency and rigorousness of the research [21]. A record was kept of the coding, themes and any changes throughout the analysis [22]. Early transcripts were read to understand whether newer codes could be applied to earlier transcripts. When it appeared that further interviews, for both the trial participants and nurses, would “not necessarily add anything to the overall story or theory” it was concluded that data saturation had been reached [23]. When the themes were finalised, the same researcher (NTM) entered the data into a matrix and summarised to allow easier comparison between trial participants, and comparisons between nurses, and to provide transparency in the coding and analysis process. Data management was facilitated through NVivo 12 Plus [24].

## Results

A total of 16 individuals were interviewed in this study, nine trial intervention participants and seven HCPs. Thirteen of the fourteen participants randomised to the trial intervention initially agreed to be contacted to participate in this interview study. Two were subsequently unavailable; one could not be contacted within the study timeframe; and one reported that they were too busy to participate, therefore nine participants were interviewed. At least one participant from five of the seven participating practices was interviewed. All nine participant interviews were conducted face-to-face in participants’ homes. The median age of participants in this report was 32 years (range 23–42 years). Eight participants lost weight during the intervention and one maintained their baseline weight (see Additional File 2). Further details of participant characteristics are reported in the trial paper previously published [13]. All nurses approached were interviewed. Four nurse interviews were conducted by telephone (three in their practice and one at home) and three were face-to-face in practices. One nurse (or GP) from each of the seven intervention practices was interviewed: six practice nurses and one general practitioner.

### Themes

Three main themes emerged from all the interview data; evaluation of the intervention, feelings around self-weighing and weight loss, barriers and facilitators to weight loss; (Additional File 3), 18 sub-themes from intervention participants and 17 sub-themes from practice nurses. This report focuses on the sub-themes where different perspectives around the same issue demonstrate disjuncture between intervention participants and nurses; or where sub-themes unique to either party contribute to contemporary discussion and debate regarding raising the topic of weight during health care consultations and routine weighing of participants by HCPs.

#### How, where and when is the right time to intervene?

No participants thought that child immunisation appointments were an inappropriate time for delivering a brief weight loss intervention, or to discuss weight with them. Some women appreciated the convenience that they did not have to make an ‘extra trip out of the house’ with their baby to be weighed, and that it was easy to integrate the intervention into their lives. Most nurses felt that the ‘ideal time’ for raising the topic of weight loss with mothers was 6–8 weeks postnatally, with some nurses suggesting an alternative or additional time could be to deliver the intervention at the six week postnatal check as this is an appointment specifically to assess the health of women, rather than the baby. There was also a feeling amongst nurses that any HCP that had contact with the mother could discuss weight loss with new mothers.

“*That would be… quite inconvenient. Just to go there to be weighed. Because I’d have to take the baby, and. In the early days… you know… It’s more difficult to get out and about, so… I was happy to do it, because I was there anyway.”* Participant 9, (lost weight)

*“I just think. as a healthcare provider I’d be more erm, open to having that discussion [about weight loss] at the postnatal check with the mum.”* Nurse 5.

Whilst most participants talked about the difficulties of caring for a new-born baby, only one would have preferred to start the intervention a few weeks later than the first immunisation; they perceived that this would place less pressure on mothers, who could be going through a “*hard*” time.

#### Feelings about self-weighing and including weighing at child immunisation appointments

No participants voiced objections to being prompted/reminded to self-weigh and record their weight on their record card. Likewise, most nurses reported that participants seemed comfortable with being weighed by them; with one nurse reporting that one participant “*jumped on the scales*” and appeared to enjoy finding out how much weight they had lost. However, two nurses reported that some of their participants were uncomfortable with being weighed, appearing embarrassed about their weight. Only one nurse reported that any of their participants had declined to be weighed by them (on these occasions participants had not lost weight). Some nurses commented that they were concerned that pressuring mothers to lose weight too soon after birth might contribute to postnatal depression (PND).

*I didn’t get the impression there were any concerns at all [about being weighed]* Nurse 3.

“*Your weight’s not going to come off straightaway. So I don’t think we should be pressurising mothers too much. And especially with things like postnatal depression*” Nurse 5.

When self-weighing, participants reported how their expectations of their weight affected their emotions; typically they felt “*good*” when expecting to see that their weight had decreased, while being worried, fearful or “*bad*” if they expected an increase in weight, due to a “*bad week*”. For some, self-weighing gave them a sense of being in control of their weight.

*“Yeah. some weeks I felt a bit anxious if I hadn’t done well,”* Participant 6, (lost weight)

“*I guess just like on days when I weigh myself and my weight has gone down, I feel great… and like, I’m in control of things. Do you know what I mean? Like I’m directing the ship and it’s going in the right direction*.” Participant 1, (lost weight)

Whilst participants were comfortable with talking about weight with their nurse at immunisations, nurses were more reticent about doing so. Most nurses were concerned about not having enough time at the appointment to discuss a sensitive, and potentially upsetting topic, and then to provide adequate support. With a new mother generally, some nurses were concerned about damaging their relationship by raising the topic. However, because participants were expecting to be weighed at the appointment as part of the intervention, nurses felt this legitimised them raising the topic.

*“In an appointment which is already quite full with the baby, you can’t offer the mum the support that you want to offer her because I’d just feel awful if somebody walked out and I was thinking ‘Oh God, I hope they’re alright’.”* Nurse 4.

Almost all participants indicated that they were able to weigh themselves most weeks and found the process easy to remember; typically doing so on a specific day. Participants found the weight displayed on the scales as an important way to see that their weight loss efforts were having an effect, and that they were making progress.

*“By just setting… the day and the time to do it meant that I could just… yeah, just every Wednesday morning.”* Participant 9, (lost weight)

#### Barriers to weighing women at child immunisations appointments

Regardless of the time allocated for immunisations at their practice, all nurses reported that there was not enough time in current appointment schedule to add more tasks such as this intervention, and a longer appointment time was required if the intervention was rolled out as part of usual care for all women in the UK. Most nurses commented that the purpose of the appointment was to vaccinate babies, therefore weighing women was not a priority.

*“I mean, it would be great… if I had time to, you know, give them the encouragement and spend more time on that, but the focus of the consultation was the child.”* Nurse 5.

#### External accountability as a strategy for weight loss

Most participants described that the intervention and the regular contact with their nurse offered a sense of accountability, of being monitored regularly and not wanting to ‘let down’ the nurse; this provided them with a source of motivation to continue to adhere to a healthy lifestyle and weight loss. In contrast, only two nurses referred to the concept of accountability when reflecting on their role in the intervention; both nurses viewed accountability as a positive feature, and that participants knowing that their past weight could be visually seen and compared to their current weight by the nurse, would result in increased motivation to lose weight. Related to this, when participants were asked how the intervention could be improved, about half thought it would be useful to have a phone number or email address to contact if they needed to ask something and did not want to wait until the next immunisation appointment. However, the nature of intervention participants’ responses suggested that this was more about a need for regular contact with a person to keep them motivated, than a need for advice per se, consistent with a desire for external accountability.

*“I think it’s more of an incentive. Especially knowing that they [the participants] was being weighed.”* Nurse 6.

*“Yes because obviously to lose weight these days… is difficult to do on your own than when you’ve got somebody there nagging you in the back of your head. You know you’ve got this programme that you’re doing. It’s like just going to Weight Watchers. You know every week you have to be weighed. It’s similar to this as well. Every week you have to be weighed, so… you’re keeping track of your weight*.” Participant 2, (no weight loss)

#### Signposting to technology for support to lose weight

Both participants and nurses commented that they felt it was acceptable to include referral to an online weight loss programme for support within child immunisation appointments. Most participants commented that the online programme was motivating. Participants expressed views that valued the online support that could be accessed frequently at any time of the day which allowed them to fit around the baby’s unpredictable schedule and they did not need to leave home to receive support. Some participants valued the fact that the information on the POWeR website came from a “*trusted source*” due to its links with the NHS. Some nurses also highlighted the problem of participants being able to access reliable advice about weight loss after pregnancy from the internet and were pleased the study website contained accurate information for women.

*“[It’s] easier because you haven’t got to go out of the house to them. You know, go out of the house with a baby in tow. Um, and you can sort of do it at home. At your own pace sort of thing”* Participant 4, (lost weight)

#### Views about HCPs providing support for weight loss to women at child immunisations

When asked who should be providing postnatal women with advice and information on how to lose weight, nurses felt that any HCP in contact with mothers would be appropriate. One nurse described their role as a nurse as ideal, since midwives or healthcare visitors were focused on the baby, not the mother. Most nurses expressed views that, at least partly, inferred weight loss was the responsibility of the mother, not them.

*“Like… people know that they need to lose weight if they are overweight.”* Nurse 5.

Several nurses felt the postnatal period was a vulnerable time and that raising the topic of weight loss was an additional “*pressure*” for women that should be avoided.

*“Just relax for a bit. You’ve been through a really traumatic experience. You might already have two toddlers running you. ragged I just think sort of give them a break really, you know?”* Nurse 4.

#### Facilitators and barriers to weight loss after giving birth

All participants were motivated to join the study because they were keen to lose weight. Several participants commented that simply being part of the trial was useful to them, a regular reminder that they had to focus on losing weight. All participants found the intervention, or some aspect of it, increased their motivation or commitment to losing weight. Most intervention participants commented on the perceived effectiveness of the intervention for them. Several participants reported that in some ways, being a new mother was a facilitator, a window of opportunity to re-start healthy habits for themselves and their family. All participants except one would recommend the study to other mothers.

“*I want to lose weight. As soon as I had a baby I wanted to lose weight because I’m planning on, a big holiday! So I want, I want to be able to go on the beach.*” Participant 2, (no weight loss)

“*We want [them] to eat healthy, you know, and it’s all vegetables and fruits that we have [them] on, so that’s nice too- We eat [their] leftovers too. They’re good for us too. We’ll show [them] like “Look, pear is good!”* Participant 1, (lost weight)

Breastfeeding was viewed both as a facilitator and a barrier. Some participants felt that it could lead to more calories being burned but others commented that it hindered their efforts to be physically active, for example when gyms did not have breastfeeding facilities and increased hunger from breastfeeding led them to eating more food.

“*[They don’]t like bottles and so swimming I can do because I can run up to [the local public swimming baths], swim for a half hour, come back and be back for [their] next feed.*” Participant 1, (lost weight)

“*The more I’m breastfeeding, I’m more hungry”* Participant 2, (no weight loss)

Most participants had to negotiate around the all the needs and schedule of their baby if they wanted to be physically active and it was difficult to leave the house to exercise. Some participants mentioned tiredness from raising a new-born baby and that this impacted their eating behaviours. Similarly, having a new-born baby meant an unknown schedule, and a necessity to plan and work around the schedule of the baby, which led to choosing less healthy foods that were quicker to prepare.

“*It’s tough… already. You have a new baby… that is depriving you of sleep. Erm, you have what, two hours sleep, interruptions overnight at times all you want is, you know, cake, wine, takeaway and a few indulgences.*” Participant 3, (lost weight)

Participants commented that maintaining behavioural changes was difficult and most discussed the temptation to “*fall off the wagon*” and to stop their weight loss efforts in the face of alternative pressures, such as stress or tiredness, or allow themselves an “*indulgence*”. The design of the study intervention was viewed as a method to help them keep “*on track*”.

***“****[Being involved] been good because it helped me to, stay in track of my weight because I see, weigh myself every week.*” Participant 2, (no weight loss)

## Discussion

In the absence of evidence to support the benefit of weight management interventions during pregnancy, postnatal interventions are increasingly important. This study explored the views of women who experienced a brief weight loss intervention delivered by nurses within routine child immunisation appointments in primary care. Whilst practice nurses reported some concerns about the timing of the intervention soon after giving birth and the time currently available to deliver the intervention with child immunisation appointments, no participants thought this was an inappropriate or unsuitable time to receive such an intervention. Nurses expressed some reservations about delivering the intervention, yet also viewed it as a useful and sustainable strategy for weight loss, as did participants. Participants and nurses felt that the intervention was easy to deliver, “*a good idea*”, and likely to increase women’s motivation for weight loss. Nurses reported that participants appeared comfortable with being weighed but some nurses had concerns about raising the topic of weight. It was felt by nurses to be a sensitive topic at a vulnerable time in women’s lives, although participants did not generally share this view.

### Weight loss after pregnancy

Studies have reported that women are motivated to lose weight after having a baby and this was also the case here where participants welcomed support from the NHS to achieve this [25, 26]. Whilst women expressed some barriers to losing weight, the time after giving birth was viewed as a good opportunity to consider other changes to their lifestyle behaviours. These views are important because such changes may have other health benefits, as physical activity is known to improve mental health such as PND, while weight loss is associated with improved mood and body image [27–30].

Overall women felt it was appropriate to offer a weight management intervention soon after childbirth. However, this finding contrasts with the views of nurses who felt that this time may not be the most appropriate time for them to offer a weight management intervention and to raise the topic of weight. Nurses felt this was a difficult time for women and they should not be burdened with any pressure to lose weight. Research has reported that there are many barriers to HCPs raising the topic of weight loss, such as not having the skills to address the problem, difficulty in raising a sensitive topic and concerns about causing offence and upsetting patients [10, 11]. It was commented by nurses that they felt more comfortable raising the topic of weight/weighing because participants were expecting to be weighed by them and because it was part of their usual care. Thus, there appears to be a disconnect between the views of nurses, and the wishes and needs of participants would like the topic of weight loss to be raised with them by their nurse. Moreover, whilst most participants discussed the difficult times of motherhood, none reported weight loss to be a sensitive topic for them to discuss with a nurse. This highlights why it is important when delivering behaviour change interventions to explore the preferences of patients/women themselves, rather than relying on the ‘expert’ opinion and/or personal beliefs of HCPs, and to actively educate HCPs about these patient preferences.

### Using child immunisation appointments to offer a weight loss intervention

Overall women felt it was appropriate to offer a weight management intervention soon after childbirth. However, this finding contrasts with the views of nurses who felt this was a difficult time for women and they should not be burdened with any pressure to lose weight. It was commented by nurses that they felt more comfortable raising the topic of weight because participation in the trial has created an expectation that this would occur, but they did not feel this was the case in routine practice. Research has reported that there are many barriers to HCPs raising the topic of weight loss, such as not having the skills to address the problem, difficulty in raising a sensitive topic and concerns about causing offence and upsetting patients [10, 11]. This research highlights a disconnect between the views of nurses, and the wishes and needs of participants, who would like the topic of weight loss to be raised with them by their nurse. This highlights why it is important when delivering behaviour change interventions to explore the preferences of patients/women themselves, rather than relying on expert opinion, and to actively educate HCPs about these patient preferences.

### Regular self-weighing as a strategy for weight loss

Participants were generally accepting of the instruction to weigh themselves regularly and to record their weight; participants reported these tasks were easy to do and remember each week. Several participants commented they had made a plan to weigh themselves at the same time and/or day each week. This is important because having an implementation strategy (action plan) is critical for long term behaviour change and preventing relapse [31]. Some researchers have suggested that feedback about body size may result in psychological distress and that regular self-weighing may negatively impact on body image and/or mood by continuously reinforcing to people that their current body size is not appropriate or ideal, or lead to unhealthy dietary behaviours, such as binge eating and skipping meals [32, 33], but there were no reports of this kind from participants. Two nurses raised concerns that the intervention might lead to an increased risk of PND, but studies, including this trial, have not found an association between self-weighing and negative psychological health [34–36, 13].

### External accountability

All the nurses perceived that the intervention would increase women’s motivation to lose weight and participants welcomed support from nurses. The principle of ‘accountability’ was the centrepiece of the intervention. It is this sense of obligation, of not “letting someone else down”, that has been identified in previous research as a “key motivating factor” in participants’ successful weight loss [37] (p.255). Whilst nurses rarely commented upon their role in providing a sense of external accountability, most participants mentioned accountability, that someone other than themselves was monitoring the progress of their weight loss efforts, or they wanted to avoid ‘letting down’ the nurse. These findings are consistent with other studies that have shown that accountability is an important strategy that facilitates health behaviour change [38, 39]. Together these findings demonstrate the usefulness to participants of including external accountability as a strategy within weight loss interventions and that more strategies are required to highlight the importance of this strategy to nurses within the training provided.

### Technology and weight loss for postnatal women

Technology is increasingly being used to assist with health behaviour change, particularly for prompting self-management of health [40]. Systematic reviews have reported that technology can increase weight loss and physical activity levels [41], and lead to more favourable weight outcomes in the postnatal period [42]. It was important for this study to capitalise on this and consider ways in which technology could be integrated into the intervention, to assist participants with self-managing their weight, but also ensuring practice nurses had an option for signposting participants for support to lose weight. No participants were frustrated or concerned about being directed to a website for support and advice about weight loss, rather than receiving support directly from a nurse. Indeed, other qualitative studies based in primary care or in the postnatal period have suggested that online weight management interventions may be acceptable to patients [43, 44]. Of note here, some participants preferred the website to support from nurses because it could be accessed more frequently from home at a convenient time. Nurses were also willing to signpost participants to the POWeR website because they felt they would be able to receive detailed support from a legitimate source.

### Strengths and limitations of the study

The findings presented here should be considered alongside some methodological strengths and limitations. We have provided in-depth experiential data from both women’s and nurses’ perspectives about a novel weight management intervention. Few studies have explored the experiences of both those delivering and receiving a weight loss intervention simultaneously. Data from dual perspectives is important because it contributes context to how the intervention was experienced and received by participants, an integral strategy when determining the feasibility and acceptability of complex interventions [45]. Women from a range of backgrounds were interviewed, and at least one participant from five of the seven intervention practices. One nurse (or GP) from each of the seven intervention practices was interviewed. A comprehensive approach to data analysis was undertaken involving four researchers with different professional backgrounds and perspectives. This research also has some limitations. The total number of individuals interviewed was modest although data saturation was reached for both populations [23]. The views reported here may represent women more motivated to lose weight. The interviews with participants were held in their homes and therefore they may have expressed more favourable views of the intervention that if they had been conducted in a neutral environment. Study participants ‘opted in’ and most lost weight, therefore, may have been more ready to consider behaviour change than those who declined the trial. The interview topic guide was shaped by the research team, who were investigating the efficacy of the intervention. However, the researcher undertaking the interviews was not involved in the design or conduct of the intervention or trial beyond data collection, analysis and writing up the findings of this study. While the researcher who conducted the interviews was experienced in conducting qualitative interviews and analysis, they had not been pregnant so may have misconstrued, or missed nuances, discussed by participants referring to this experience.

## Conclusions

Embedding a brief weight management intervention into child immunisation appointments presents an opportunity to routinely identify and offer support to women with overweight/obesity. Overall participants reported the intervention was acceptable to them, it was convenient, and they welcomed the support to lose weight that was offered. Whilst nurses, expressed concerns about raising the topic of weight in the early postnatal period, they felt the intervention was easy to deliver and that it would help to motivate women to lose weight.

## Supplementary Information

**Additional file 1.** Interview schedules for trial participants and practice nurses.

**Additional file 2.** Participants demographics.

**Additional file 3. **List of themes for participants and practice nurses.

## Data Availability

The datasets used and analysed during this study are available from the corresponding author on reasonable request. Access to anonymised data may be granted following review of the request. Exclusive use will be retained until the publication of major outputs.

## Abbreviations

BMI: Body mass index
BWH: Birmingham Women’s Hospital
KG: Kilograms
HCPs: Healthcare professionals
GP: General practitioner
NHS: National Health Service
RCT: Randomised controlled trial
POWeR: Positive Online Weight Reduction
UK: United Kingdom
