# Supplementary material for: The experiences of postnatal women and healthcare professionals of a brief weight management intervention embedded within the national child immunisation programme
Source: BMC Pregnancy Childbirth. 2021 Jun 29;21:462. doi: 10.1186/s12884-021-03905-3 (PMC8243541; doi:10.1186/s12884-021-03905-3)
Supplement: Supplementary file 1 — Additional file 1. Interview schedules for trial participants and practice nurses. [file 12884_2021_3905_MOESM1_ESM.docx]

# **Additional File 1: Interview schedules for trial participants and practice nurses**

Warm up question

How old is your baby now? How’s it all going?

Pre-pregnancy weight maintenance

Now I’d like to talk about any previous weigh loss attempts you made before your last pregnancy.

- Before joining this trial, had you ever tried to lose weight?
- What type of things did you do to try and manage your weight in the past?

Reasons for participating in trial

- Can you tell me about what it’s been like for you being involved in the PIMMS-WL trial?
  - What were you hoping to get out of being a part of PIMMS-WL?
  - Why did you want to take part?

Self-weighing

- Could you tell me what you thought about having to weigh yourself once a week?
- Can you tell me how weighing yourself made you feel?
- How useful did you find weighing yourself regularly?
- Were you able to weigh yourself once a week?
- Was it easy to remember to weigh yourself regularly?
- Can you tell me how important the number on the scales was for you and why?

Current weight management

- Are you still attempting to manage your weight?
- Can you tell me a bit about what you do to manage your weight presently?
- What do you use to help you measure/gauge your progress?

Immunisation appointments

I would now like to talk about what happened during baby’s immunisation appointments.

- Could you describe a typical immunisation appointment? Could you walk me through what would happen during these appointments?
- You mentioned that the nurse….did the nurse ask if she could weigh you during these appointments?
- How did you feel when the nurse was weighing you?
- What did you think of having the nurse weigh you during your baby’s immunisation appointment?
- Could you tell me a little bit about what the nurse said to you while she was weighing you?
- Did the nurse hold your baby while you were on the scales?
- Can you tell me how you felt knowing that you would be weighed at your baby’s immunisation appointments?

POWeR website

Now I want to ask you about the POWeR website.

- Were you able to access the site?
- Can you tell me what sort of things you looked at on the website?
- What did you think of the website?
- What did you think about being referred to a website for weight management advice instead of being offered it during the immunisation appointment?

Additional questions

- Now that you’ve been involved in the PIMMS-WL trial, how do you feel about managing your weight?
- Can you tell me what sort of things we could have done differently to make things easier for you?
- Can you tell me what would you think we should have done to make being involved in the PIMMS-WL trial better for you?
- At the start of this study you completed a consent form to agree to participate.  This means that only women like you who signed this consent form can take part and the nurse will only weigh women who filled in the consent form.  In the next study we are thinking about not having a consent form and the nurse just weighing every woman who comes to each of the immunisation appointments and writing this in the red book as part of routine care after having a baby.  The nurse would also encourage every woman to weigh themselves each week like we asked you to do. How do you think women would react to this?  Or how would they feel about it?

Prompt:  Women could still refuse to be weighed it is just that the nurse will expect to do it routinely in all women unless they object – rather than having a consent form like you did?

- What sort of things do you think would help women lose weight after pregnancy?
- Would you recommend the trial to your friends or other new mums?
- Is there anything else you’d like to tell me?

**Nurses interview schedule**

## Warm up

- How long have you been a practice / research nurse?
- How long have you been giving immunisations for?

## General information

- Who usually brings the baby to these immunisation appointments?
- Since you’ve been working with new mums, what sort of impact do you think being overweight or obese has had on them?
- When you meet new mums at these appointments, is the topic of weight and weight management commonly raised?

## Nurses training

Now I’d like to talk about the nurses training you received on how to deliver the intervention during the immunisation appointments.

- Could you describe the training you received for this trial?
- What did you think of the training so far?
- How did you find the nurses’ training manual?
- How often do you think you referred back to it?
- What do you think we could have added or changed about the training sessions to make them more effective for you?
- After the training, how prepared did you feel to deliver the intervention?

Immunisation appointments

I would now like to switch topics a little and talk about what happened during baby immunisation appointments with mothers involved in the PIMMS-WL trial.

- How easy was it to identify mothers who were taking part in the trial?
- Could you describe a typical immunisation appointment with a PIMMS-WL mum? Could you walk me through what would happen during these types of appointments?
- Can you tell me how you felt knowing that you would be asking to weigh mothers involved in the trial?
  - How comfortable did you feel?
  - How confident did you feel?
- Could you tell me a bit about what sort of things you’d say to the mum while you were weighing her?
  - How comfortable do you think the mothers were with you weighing them?
  - Did any women refuse to be weighed?
  - What sorts of reasons did they give for not wanting to be weighed? Did any delay or seem reluctant to get on the scales?
- What sort of reactions did you get from mothers who attended these appointments alone compared to those who attended with their partners or mothers?
- Where did the baby get put while the mothers were on the scales and you were recording their weight?
  - Were there practical issues to consider when having to weigh the mothers?
- What did you think of having to record their weight in the red book?
  - Was it easy to remember?
  - Was the weight record card easily accessible?
- How much more time did appointments take when you had to weigh the mothers?
- Can you tell me what you think it would be like if you had to weigh every mother you saw during your baby immunisation clinics?

## POWeR website

Now I want to ask you about the POWeR website.

- Can you tell me what sort of responses you received when you referred mothers who asked you for weight loss advice to the POWeR website?
- Did any of the mothers mention the POWeR website? Did they bring it up?
- Can you tell me a bit about what they said?
- Were others more positive / negative about it?
- Were you able to have a look at the POWeR website?
  - What did you think of the website?
- What did you think about referring mothers to a website for weight management advice during the immunisation appointment?

## Additional questions

- Before taking part in this study, what did you tell mothers who asked you for weight loss advice?
- When do you think is the ideal time to try and encourage new mums to start thinking about trying to lose / manage their baby weight?
- What sort of things do you think would help women lose weight after pregnancy?
- Who do you think should be providing mothers with this advice?
- Can you tell me what sort of things we could have done differently to make things easier for you?
- Can you tell me what would you think we should have done to make being involved in the PIMMS-WL trial better for you?
- Is there anything else you’d like to tell me?
