# Supplementary material for: The experiences of postnatal women and healthcare professionals of a brief weight management intervention embedded within the national child immunisation programme
Source: BMC Pregnancy Childbirth. 2021 Jun 29;21:462. doi: 10.1186/s12884-021-03905-3 (PMC8243541; doi:10.1186/s12884-021-03905-3)
Supplement: Supplementary file 2 — Additional file 2. Participants demographics. [file 12884_2021_3905_MOESM2_ESM.docx]

# **Additional file 2: Participants demographics**

| **Participants characteristics (n=9)** | |
| --- | --- |
| **Age (years)** | |
| 20 < 25 | 1 |
| 25 < 30 | 1 |
| 30 < 35 | 4 |
| 35 < 40 | 1 |
| 40 < 45 | 2 |
| **Ethnicity** |  |
| White | 5 |
| Black African | 1 |
| Other | 3 |
| **Number of children** | |
| 1 | 3 |
| 2 | 4 |
| 3 | 2 |
| **Baseline body mass index** | |
| 27.5 < 30 | 3 |
| 30 < 32.5 | 3 |
| 32.5 < 35 | 1 |
| 35 < 37.5 | 1 |
| 37.5 < 40 | 0 |
| 40 < 42.5 | 1 |
| **Marital status** | |
| Married | 6 |
| Single (living with partner) | 2 |
| Single (living alone) | 1 |
| **Self-reported financial status (employment status)** | |
| I have enough money if I plan my spending carefully (in paid employment) | 3 |
| I have enough money if I plan my spending carefully (looking after the home/family) | 1 |
| I normally have enough money for whatever I want (in paid employment) | 3 |
| I normally have enough money for whatever I want (looking after the home/family) | 1 |
| I normally have enough money for whatever I want (student) | 1 |
| **GP practice (IMD rank)** | |
| High | 8 |
| Medium | 1 |
| Low |  |
