# Supplementary material for: The experiences of postnatal women and healthcare professionals of a brief weight management intervention embedded within the national child immunisation programme
Source: BMC Pregnancy Childbirth. 2021 Jun 29;21:462. doi: 10.1186/s12884-021-03905-3 (PMC8243541; doi:10.1186/s12884-021-03905-3)
Supplement: Supplementary file 3 — Additional file 3. List of themes for participants and practice nurses. [file 12884_2021_3905_MOESM3_ESM.docx]

# **Additional file 3: List of themes for participants and practice nurses**

| **Mothers coding framework** | **Nurses coding framework** |
| --- | --- |
| EVALUATION OF THE INTERVENTION | |
| Areas for improvement | Areas for improvement |
| Credibility of the weighing scales |  |
|  | Assessment of training |
|  | Identifying intervention participants |
|  | Impact of the study intervention on appointment |
| Impact of the study/intervention | Impact of the intervention on mothers |
| Importance of numbers |  |
| Opt Out | Rolling out the intervention |
| Positives of the study/intervention | Positives of the intervention |
|  | Recording weight |
| Steps in appointment | Steps in appointment |
| Website content | Website |
| Website positives |  |
| FEELINGS AROUND WEIGHING AND WEIGHT LOSS | |
| Accountability - mothers |  |
| Emotional issues around losing weight |  |
| Feelings knowing to be weighed |  |
| Feelings when nurse weighs | Mothers’ feelings while weighing |
| Reasons for starting the study |  |
| Self-weighing feelings | Nurses’ feelings when weighing mothers |
| BARRIERS AND FACILITATORS TO WEIGHT LOSS | |
| Barriers to weight loss | Perceived barriers in mothers to weight loss |
| Facilitators for weight loss | Perceived facilitators in mothers to weight loss |
| How to lose weight | How to lose weight |
|  | Ideal time, space and role for weight loss and intervention |
|  | Potential barriers in nurses to weight loss |
